# Association between HbA1c and hearing loss: a tertiary care center-based study

**DOI:** 10.1038/s41598-023-44909-7

**Published:** 2023-10-27

**Authors:** Hyun Jin Lee, Sung Goo Yoo, Sun Jung Lee, Jae Sang Han, In Young Choi, Kyoung Ho Park

**Affiliations:** 1 Department of Otorhinolaryngology-Head and Neck Surgery, Incheon St. Mary’s Hospital, College of Medicine, The Catholic University of Korea, Seoul, Republic of Korea; 2 Department of Medical Informatics, College of Medicine, The Catholic University of Korea, 222 Banpo-daero, Seocho-gu, Seoul, 06591 Republic of Korea; 3 Department of Otolaryngology-Head & Neck Surgery, Seoul St. Mary’s Hospital, College of Medicine, The Catholic University of Korea, Seoul, Korea

**Keywords:** Diseases, Endocrinology, Health care, Medical research

## Abstract

The purpose of this study was to investigate the correlation between glycated hemoglobin (HbA1c) levels and hearing loss (HL) using data from a tertiary hospital. Our hypothesis regarding the relationship between HL and HbA1c levels was that elevated HbA1c levels are associated with an increased risk of HL. We retrospectively reviewed the medical charts of patients diagnosed with sensorineural HL or diabetes between 2006 and 2021 at the Catholic Medical Center (CMC). Data were collected from the CMC’s Clinical Data Warehouse. Participants were selected from patients who were prescribed pure-tone audiometry and an HbA1c blood test. The survey was completed for 5287 participants. The better ear pure-tone audiometry (PTA) for air conduction thresholds at 500, 1000, 2000, and 4000 Hz was calculated. Sensorineural HL was defined as a better ear PTA of 25 dB or higher. We used the HbA1c level as a diagnostic criterion for diabetes. The following criteria were used to define the HbA1c level: normal, HbA1c level below 5.6%; prediabetes, level between 5.6 and 6.4%; and diabetes, level of 6.5% or more. Among 5287 participants, 1129 were categorized as normal, 2119 as prediabetic, and 2039 as diabetic. The diabetic group was significantly older (*p* < 0.05). The PTA also significantly deteriorated in the diabetes group (*p* < 0.05). We analyzed the effects of age, sex, and HbA1c level on frequency-specific hearing using multiple regression. The hearing thresholds at all frequencies deteriorated significantly with increasing age and HbA1c level (*p* < 0.05). A case–control study was also performed to facilitate a comprehensive comparison between distinct groups. The participants were categorized into two groups: a case (PTA > 25 dB) and control group (PTA ≤ 25 dB), based on their PTA threshold of four frequencies. After adjusting for age and sex, we found no significant odds ratio (OR) of HL between the prediabetes group and the normal group. Notably, the OR of HL was significantly higher in the diabetes group with each PTA threshold and frequency. The 6.3% HbA1c level cutoff value was determined by analyzing the receiver operating characteristic curve for predicting hearing impairment > 25 dB. Diabetes was associated with hearing loss in all frequency ranges, particularly at high frequencies. Screening for HL is strongly recommended for patients with elevated HbA1c levels.

## Introduction

Sensorineural hearing loss (SNHL) has a negative impact on both social communication and personal functional ability and is substantially linked to a lower quality of life^1^. SNHL impacts social and emotional function, communication, and cognitive function, and can lead to depression, resulting in a variety of comorbidities^2,3^. Hearing loss (HL) among adults increases with age and differs between countries. In the United States, approximately half of people in their 60 s (60–69 years old) and 80% of people aged 85 years or older are categorized as having HL using the pure-tone audiometry (PTA) average of the National Health and Nutrition Examination Survey (NHANES)^4,5^. The prevalence of HL in the South Korean population has been collected from the Korea-NHANES, where approximately 36% of individuals in their 60 s (60–69 years old) and 66% in their 70 s (70–79 years old) have HL based on the PTA^6^. Age-related changes in the auditory system are the primary causes of HL in adults. In addition to the degenerative effects of aging on the cochlea, chronic diseases cause HL in older individuals^7^.

There are other factors that affect HL besides age. Better diet quality was associated with better high-frequency hearing thresholds, according to cross-sectional NHANES data from 2366 adults aged 20 to 69 years^8^. Studies on adult populations have provided evidence for interactions between specific foods, oxidative and inflammatory processes, and HL^9,10^. Obesity, diabetes, and cardiovascular disease are currently some of the “lifestyle diseases” that have been related to unhealthy lifestyles. The presence of these diseases has been associated with an increased risk of HL in several studies^11–13^.

Globally, diabetes mellitus (DM) is a leading cause of morbidity, disability, and mortality, with a steadily increasing incidence and prevalence^14^. In 2020, the estimated prevalence of diabetes among Korean adults aged 30 years and above was 16.7%^15^. DM is a metabolic disease that causes complications in vascular and neurological function. Prediabetes and type 2 diabetes are diagnosed based on blood glucose levels and glycated hemoglobin (HbA1c). Prediabetes is a medical condition in which blood sugar levels are elevated but not high enough to be diagnosed as type 2 diabetes. Diabetes is diagnosed at an HbA1c level greater than or equal to 6.5%, fasting blood glucose level greater than or equal to 126 mg/dL, and a two-hour blood glucose level greater than or equal to 200 mg/dL^16^. DM has been associated with a higher prevalence of HL. Cochlear microangiopathy, perilymph hyperglycemia, and neuropathy have been implicated in the pathogenesis of DM-associated HL^17,18^.

There have been many reports on the correlation between diabetes and HL. Controlling for potential covariates, a previous large population-based study showed an association between diabetes and HL (odds ratio [OR] 1.41, 95% confidence interval [CI] 1.05–1.88)^19^. Kakarlapudi et al.^20^ suggested that SNHL was more prevalent in patients with diabetes than in a control group of patients without diabetes. In addition, Kim et al.^21^ showed that the risk of HL increased if the HbA1c level was greater than 5% in patients with diabetes compared with that of patients with prediabetes, also suggesting that diabetes is associated with a significant risk of HL. Another study found an association between HbA1c levels and hearing impairment in participants without diabetes^1^.

The study hypothesis regarding the relationship between HL and HbA1c levels was that elevated HbA1c levels are associated with an increased risk of HL. Diabetes has been suggested to be associated with a variety of hearing abnormalities. Consequently, the present study analyzed the correlation between HbA1c levels and HL using a large-scale study using clinical data from a tertiary hospital.

## Materials and methods

### Study population

**Figure 1:**
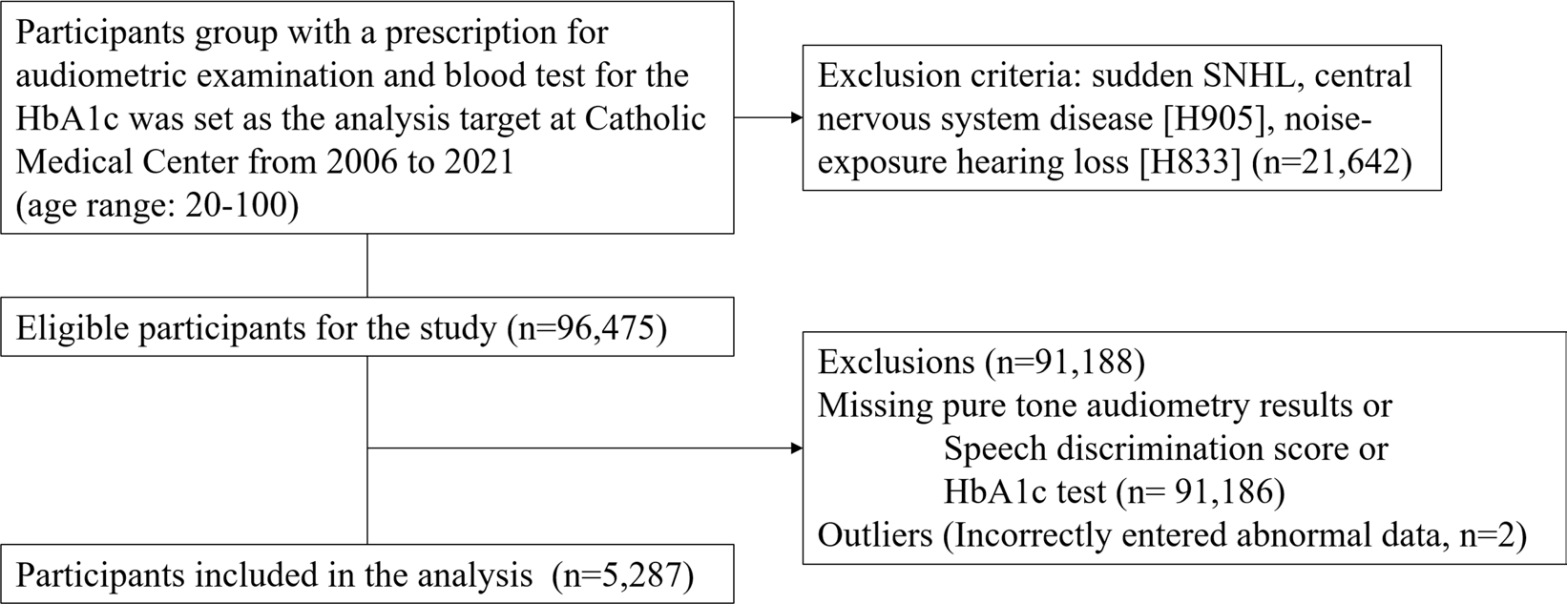
A flow diagram of the study design. *HbA1c* glycated hemoglobin, *SNHL* sensorineural hearing loss.

We conducted a retrospective medical chart review of patients diagnosed with SNHL or diabetes between 2006 and 2021 at the Catholic Medical Center (CMC). Data were collected from the CMC’s Clinical Data Warehouse. Participants were selected from patients who were prescribed an audiometric examination and an HbA1c blood test. PTA and speech audiometry (SA) were performed on both ears. HbA1c levels were also evaluated in all included participants. The exclusion criteria were as follows: (1) age < 20 years; (2) sudden SNHL; (3) central nervous system disease, including stroke, cerebrovascular disease, and trauma; (4) noise-exposure HL; and (5) inner ear disease or malformation. The International Classification of Diseases Tenth Revision codes H905 and H833 were employed as filters to identify cases of sudden HL, noise exposure-related HL, and HL resulting from trauma. The number of eligible participants for the study was 96,475. We further excluded patients with missing audiometric test results, speech discrimination scores (SDSs), and HbA1c tests, as well as outliers (results outside the normal range). Finally, the survey was completed for 5287 participants (Fig. 1).

This study was approved by the Institutional Review Board of the Catholic University of Korea, Seoul St. Mary’s Hospital (No. KC21WISI0962) and followed the tenets of the Declaration of Helsinki. Patient consent was waived and did not adversely affect the rights and welfare of the subjects. A waiver of informed consent from all of the patients was approved by the Institutional Review Board of the Catholic University of Korea, Seoul St. Mary’s Hospital.

### Audiometric measurements

PTA and SA were evaluated. A GSI 61TM audiometer (Grason-Stadler, Inc., St. Eden Prairie, MN, USA) was used for audiometric testing in a soundproof booth and was calibrated according to the American National Standard Institute guidelines (S3.6–1996). Pure-tone air (250–8000 Hz) and bone conduction (250–4000 Hz) thresholds were measured using clinical audiometers in a double-walled audio booth. The mean PTA thresholds for air conduction at 500, 1000, 2000, and 4000 Hz [PTA_4_ = (threshold at 500 Hz + 1000 Hz + 2000 Hz + 4000 Hz)/4] were calculated. SNHL was defined as a PTA threshold of 25 decibels (dB) or higher. In cases where the hearing thresholds were asymmetrical, the ear threshold with better hearing was used for the analysis. With this step, some participants with pathologically damaged hearing could be excluded. Participants with unilateral hearing loss were classified as the “normal” PTA group. We used the criteria established by the World Health Organization (WHO) to classify the extent of HL in affected individuals^22^.

The speech recognition threshold (SRT) was defined as the level at which patients heard and correctly repeated the spondaic words 50% of the time. Each spondee had two Korean syllables with equal stress placed on each. A word recognition test was performed to obtain word recognition scores, which were measured at the most comfortable hearing level using 50 monosyllabic Korean words heard in everyday life. Korean words were taken from a validated and standardized resource (Hahm’s list) and were phonetically balanced^23^. A trained audiometric technician performed the speech audiometric test in a dedicated acoustic detection booth^23^. The sound was provided at a constant intensity through a microphone.

### Assessment of diabetes

We used the HbA1c level as a diagnostic criterion for diabetes. HbA1c reflects the average blood glucose level over the preceding 8–12-week period (the lifespan of red blood cells) and is used as an indicator of glycemic control^24^. The following criteria were used to define the HbA1c level: normal, HbA1c level below 5.6%; prediabetes, level between 5.6 and 6.4%; and diabetes, level of 6.5% or more.

### Statistical analysis

All statistical analyses were performed using R software (R Core Team, Vienna, Austria). In the descriptive analysis of the survey data, arithmetic means and percentages with standard errors for estimates were calculated. Two-sample t-tests and chi-squared tests were used to compare the groups. Multiple regression analysis was performed to test the association between HL and the HbA1c level. Statistical significance was set at *p* < 0.05. Case–control analysis was conducted to facilitate a rigorous comparison and evaluation of the results seen in the experimental group. The case group consisted of patients with HL, namely those with a PTA threshold of 25 dB or higher. In contrast, the control group was comprised of individuals without HL, characterized by a PTA threshold of 25 dB or better.

To assess the risk factors associated with HL in individuals with diabetes, univariate and multivariate logistic regression analyses were conducted to calculate the OR and 95% CI. The receiver operating characteristic (ROC) curve was used to compare the true-positive rate (sensitivity) to the false-positive rate (1 − specificity). This graph is helpful in diagnostic studies to evaluate the ability of biomarkers or diagnostic tests to discriminate between different conditions^25^. The positive likelihood ratio, also known as LR + (sensitivity/1 − specificity), expresses the degree to which positive outcomes are significantly more common among individuals with hearing impairment than among those without the disease^26^. This study used the R software multiple ROC package (R Core Team) to obtain the optimal cut-off value.

## Results

### Basic characteristics

The baseline characteristics of the study population are summarized in Table 1. Of the 5287 participants, 1129 were classified as normal, 2119 as prediabetic, and 2039 as diabetic. The diabetic group was significantly older (*p* < 0.05). The SDS results were significantly poorer in the diabetes group than in the other groups (*p* < 0.05). The better ear PTA (500, 1000, 2000, and 4000 Hz) was also significantly decreased in the diabetes group (*p* < 0.05). Hearing impairment was classified according to the WHO standards (normal and slight, moderate, severe, and profound impairment). With worsening HL, age and HbA1c were significantly increased (*p* < 0.05) and SDS was significantly decreased (*p* < 0.05) (Table 2).

**Table 1:** Clinical characteristics of subjects according to HbA1c.

Parameter	Total subjects (n = 5287)	Normal (n = 1129)	Prediabetes (n = 2119)	Diabetes (n = 2039)	*p*-value
Age (years)	68.92 ± 12.52	64.4 ± 15.1	69.9 ± 11.3	70.4 ± 11.5	< 0.001***
Sex					
Male	2419 (45.8%)	519 (46.0%)	898 (42.4%)	1002 (49.1%)	< 0.001***
Female	2868 (54.2%)	610 (54.0%)	1221 (57.6%)	1037 (50.9%)	< 0.001***
HbA1c (%)					
Mean (SE)	6.4 ± 1.1	5.3 ± 0.3	6.0 ± 0.2	7.5 ± 1.1	< 0.001***
SDS (%)	84.3 ± 23.9	86.0 ± 23.3	84.1 ± 23.2	81.3 ± 24.8	< 0.001***
PTA (dB)	29.17 ± 17.63	25.5 ± 17.4	28.9 ± 17.3	31.5 ± 17.8	< 0.001***

*HbA1c* glycated hemoglobin, *SDS* speech discrimination score, *PTA* pure-tone audiometry (500, 1000, 2000, 4000 Hz) of the better ear, *SE* standard error.

***p < 0.001.

**Table 2:** Clinical characteristics of subjects according to WHO hearing classification.

Parameter	Normal (n = 2681)	Slight (n = 1319)	Moderate (n = 997)	Severe (n = 227)	Profound (n = 63)	*p*-value
Age (years)	63.5 ± 12.3	72.7 ± 9.2	76.6 ± 9.7	76.9 ± 12.0	70.4 ± 13.2	< 0.001***
Sex						
Male	1125(42.0%)	630 (47.8%)	500 (50.2%)	128 (56.4%)	36 (57.1%)	< 0.001***
Female	1556(58.0%)	689 (52.2%)	497 (49.8%)	99 (43.6%)	27 (42.9%)	< 0.001***
HbA1c (%)	6.3 ± 1.1	6.5 ± 1.1	6.6 ± 1.2	6.7 ± 1.3	6.6 ± 1.2	< 0.001***
SDS (%)	96.4 ± 9.9	84.8 ± 13.4	62.2 ± 23.3	34.0 ± 28.6	16.2 ± 27.8	< 0.001***

*HbA1c* glycated hemoglobin, *SDS* speech discrimination score.

### Association between HL and HbA1c

We analyzed the effects of age, sex, and HbA1c level on frequency-specific hearing using multiple regression (Table 3). The hearing thresholds at all frequencies deteriorated significantly with increasing age and HbA1c level (*p* < 0.05). At all frequencies except 500 Hz, the hearing threshold of males was significantly poorer than hearing thresholds for females (*p* < 0.001).

**Table 3:** Multiple regression analysis for factors affecting each hearing frequency.

	Age	Sex (Male)	HbA1c	Adjusted R^2^
	Coefficient (95% CI)	Coefficient (95% CI)	Coefficient (95% CI)	Sig. F
500 Hz	0.05 (0.47–0.53)	0.55 (− 0.28 to 1.38)	1.21 (0.84–1.59)	0.153
*p*-value	< 0.001***	0.193	< 0.001***	< 0.001***
1000 Hz	0.59 (0.56–0.63)	1.22 (0.34 to 2.10)	1.10 (0.70–1.50)	0.178
*p*-value	< 0.001***	0.001**	< 0.001***	< 0.001***
2000 Hz	0.67 (0.63–0.70)	3.36 (2.44 to 4.29)	1.08 (0.66–1.50)	0.205
*p*-value	< 0.001***	< 0.001***	< 0.001***	< 0.001***
4000 Hz	0.82 (0.78–0.87)	12.21 (11.15 to 13.28)	1.37 (0.89–1.85)	0.269
*p*-value	< 0.001***	< 0.001***	< 0.001***	< 0.001***
8000 Hz	1.11 (1.06–1.15)	9.60 (8.44 to 10.77)	1.41 (0.88–1.94)	0.316
*p*-value	< 0.001***	< 0.001***	< 0.001***	< 0.001***

*HbA1c* glycated hemoglobin, *CI* confidence interval.

***p < 0.001; **p < 0.01.

A case–control study was performed to facilitate a comprehensive comparison between distinct groups. The participants were categorized into two groups: a case group and a control group, based on their PTA threshold of four frequencies. Table 4 presents an overview of the demographic characteristics, HbA1c levels, and SDSs for the two groups of patients. Univariate and multivariate logistic regression analyses were employed to assess the degree of correlation between postulated risk factors related to diabetic conditions and the probability of developing HL for each PTA threshold and frequency. The HbA1c level was stratified into three groups as discussed earlier in the text: normal, prediabetes, and diabetes. The normal group (control) was designated as a reference group. The univariate logistic regression analysis revealed that the crude OR for HL was approximately 1.5 times higher in the prediabetes group and two times higher in the diabetes group compared with those in the normal group, across all frequency bands. Nevertheless, after adjusting for age and sex, there was no significant OR of HL between the prediabetes group and the normal group. Notably, the OR of HL was significantly higher in the diabetes group with each PTA threshold and frequency. The adjusted odds ratio for hearing loss in the group of individuals with diabetes was 1.28 (95% confidence interval 1.25–1.75) in the 500 Hz frequency range. The diabetic group exhibited an adjusted odds ratio of 1.35 (CI 1.10–1.65) for hearing loss specifically in the 8000 Hz frequency range. A greater odds ratio was detected in the high frequency band compared to the low frequency band (Table 5).

**Table 4:** Characteristics of the patients in the case–control study.

Parameter	Case group (n = 2606)	Control group (n = 2681)	*p*-value
Age (years)	74.5 ± 10.0	63.5 ± 12.3	< 0.001***
Sex			
Male	1294 (49.7%)	1125 (42.0%)	< 0.001***
Female	1312 (50.3%)	1556 (58.0%)	< 0.001***
HbA1c (%)	6.6 ± 1.1	6.3 ± 1.1	< 0.001***
SDS (%)	70.0 ± 26.5	96.4 ± 9.9	< 0.001***

Case group, pure-tone audiometry (PTA) average > 25 dB; Control group, PTA average ≤ 25 dB.

*HbA1c* glycated hemoglobin, *SDS* speech discrimination score.

***p < 0.001.

**Table 5:** Risk of hearing loss according to diabetic condition (HbA1c level).

		Case group	Control group	Crude OR	Adjusted OR*
PTA		2606	2681		
Diabetic status (HbA1c)	Normal	693 (25.8%)	436 (16.7%)	1 (ref)	1 (ref)
	Prediabetes	1088 (40.6%)	1031 (39.6%)	1.51 (1.30–1.75)^a^	1.12 (0.95–1.11)^NSa^
	Diabetes	900 (33.6%)	1139 (43.7%)	2.01 (1.73–2.33)^a^	1.47 (1.25–1.75)^a^
PTT 500 Hz		1564	3723		
Diabetic status (HbA1c)	Normal	263 (16.8%)	866 (23.3%)	1 (ref)	1 (ref)
	Prediabetes	603 (38.6%)	1516 (40.7%)	1.31 (1.11–1.55)^b^	1.12 (0.95–1.11)^NSb^
	Diabetes	698 (44.6%)	1341 (36.0%)	1.71 (1.42–2.02)^a^	1.28 (1.07–1.53)^c^
PTT 1000 Hz		2003	3284		
Diabetic status (HbA1c)	Normal	340 (17.0%)	789 (24.0%)	1 (ref)	1 (ref)
	Prediabetes	803 (40.1%)	1316 (40.1%)	1.42 (1.21–1.65)^a^	1.06 (0.89–1.26)^NSc^
	Diabetes	860 (42.9%)	1179 (35.9%)	1.69 (1.45–1.98)^a^	1.24 (1.05–1.47)^a^
PTT 2000 Hz		2320	2967		
Diabetic status (HbA1c)	Normal	397 (17.1%)	732 (24.7%)	1 (ref)	1 (ref)
	Prediabetes	927 (40.0%)	1192 (40.2%)	1.43 (1.24–1.67)^a^	1.06 (0.90–1.10)^NSd^
	Diabetes	996 (42.9%)	1043 (35.2%)	1.76 (1.52–2.05)^a^	1.28 (1.08–1.51)^a^
PTT 4000 Hz		3311	1976		
Diabetic status (HbA1c)	Normal	590 (17.8%)	539 (27.3%)	1 (ref)	1 (ref)
	Prediabetes	1315 (39.7%)	804 (40.7%)	1.49 (1.29–1.73)^a^	1.08 (0.91–1.28)^NSe^
	Diabetes	1406 (42.5%)	633 (32.0%)	2.03 (1.75–2.36)^a^	1.42 (1.19–1.69)^a^
PTT 8000 Hz		4206	1081		
Diabetic status (HbA1c)	Normal	329 (30.4%)	800 (19.0%)	1 (ref)	1 (ref)
	Prediabetes	431 (39.9%)	1688 (40.1%)	1.61 (1.36–1.90)^a^	1.00 (0.82–1.22)^NSf^
	Diabetes	321 (29.7%)	1718 (40.8%)	2.20 (1.85–2.62)^a^	1.35 (1.10–1.65)^d^

*PTA* pure-tone audiometry (average) (500, 1000, 2000, 4000 Hz), *OR* odds ratio, *HbA1c* glycated hemoglobin, *PTT* pure-tone threshold.

^a^*p*-value < 0.001, ^b^*p*-value = 0.002, ^c^*p*-value = 0.007, ^d^*p*-value = 0.005.

^NSa^*p*-value = 0.193, ^NSb^*p*-value = 0.924, ^NSc^*p*-value = 0.496, ^NSd^*p*-value = 0.462, ^NSe^*p*-value = 0.462, ^NSf^*p*-value = 0.962.

*Multivariate logistics regression analysis adjusted for age and sex.

**Figure 2:**
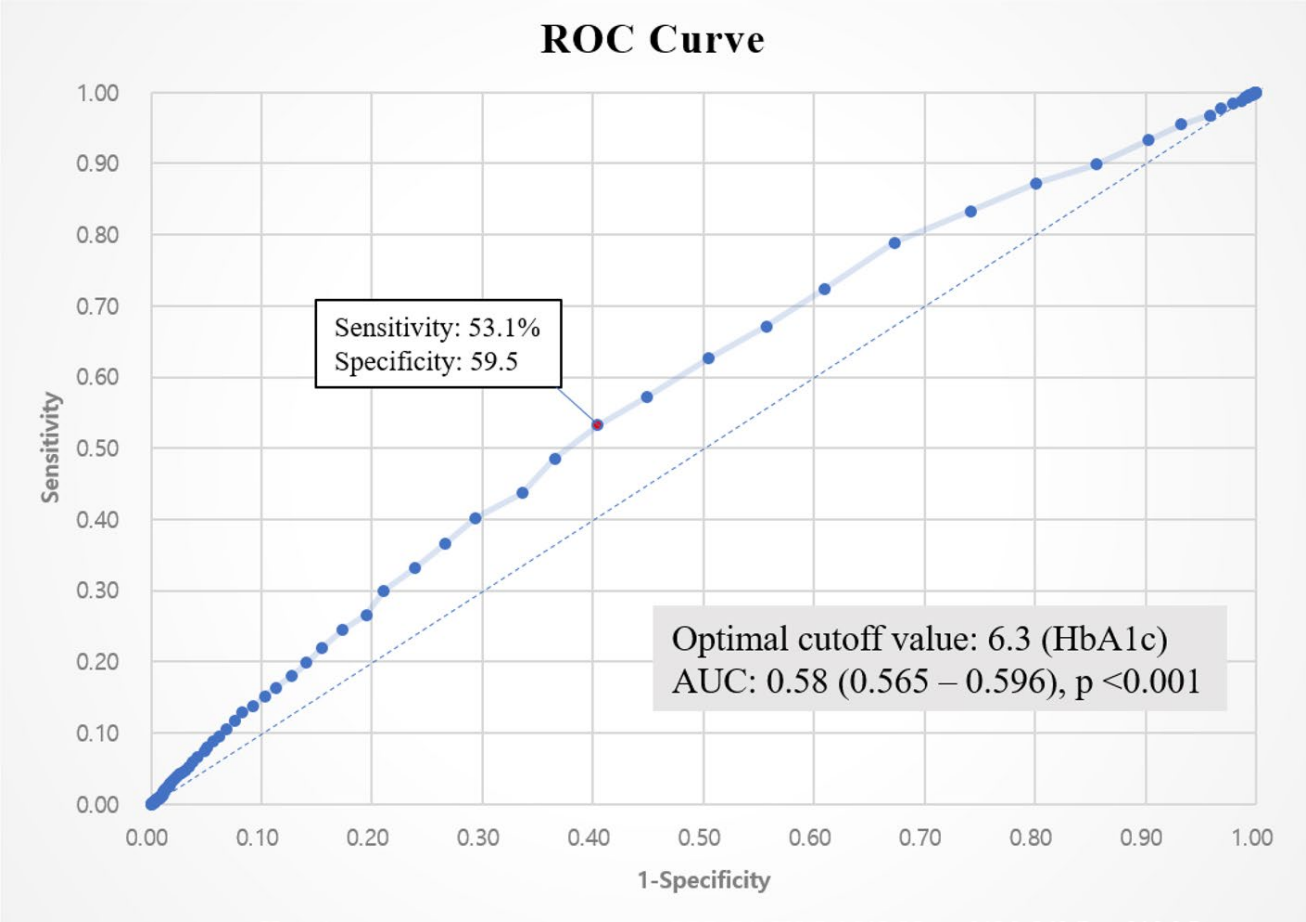
ROC curve (hearing loss prediction model based on HbA1c level). *ROC* receiver operating characteristic, *HbA1c* glycated hemoglobin, *AUC* area under the curve.

Finally, we analyzed the cutoff value of the HbA1c level that can predict HL. The cutoff value of 6.3% HbA1c was determined by analyzing the ROC curve for predicting hearing impairment > 25 dB, with the best sensitivity (53.1%) and specificity (59.5%) (*p* < 0.05). The area under the curve was 0.58 (Fig. 2).

## Discussion

Our study revealed a correlation between HL and HbA1c. This cohort study included both inpatient and outpatient data obtained from tertiary hospitals. We consider the tertiary hospital-based nature of this study as a strength, as tertiary hospitals provide healthcare from specialists in a large hospital setting after referral from providers of primary care and secondary care in smaller facilities. Notably, the hearing threshold of the definite diabetes group was significantly poorer than that of the normal and prediabetes groups at all frequencies. Age is known to have an impact on the degree of HL. Therefore, we adjusted for age and found that the diabetes group experienced a higher degree of HL than the non-diabetes group. We demonstrated that HL was associated with HbA1c levels of 6.3% or higher.

HbA1c is a type of hemoglobin commonly used to measure blood sugar levels^27^. Our hypothesis regarding the association between HL and HbA1c levels was that high levels of HbA1c can lead to a higher risk of HL. Hearing impairment resulting from high HbA1c levels is caused by damage to the small blood vessels in the inner ear. Microangiopathy, which is prevalent in patients with diabetes, affects multiple organ systems and may affect the inner ear vessels, such as the labyrinthine artery^28^. When chronic or temporary microvascular disorders occur in the inner ear, pathophysiological changes lead to cochlear microcirculatory dysfunction and HL^29^. Additionally, diabetes can damage the auditory nerve, leading to HL^30^. Insulin system dysfunction can affect the entire body. The sensory receptors and supporting cells of the cochlea, stria vascularis, and spiral ligament contain insulin receptors, glucose transporters, and insulin signaling components, making hearing and balance vulnerable to glucose utilization deficits^31,32^. Hyperglycemia also causes a number of functional diseases, such as those related to mitochondrial deoxyribonucleic acid damage^33^. Affected mitochondria can inhibit oxidative phosphorylation and adenosine triphosphate production, leading to failure in energy-intensive organ systems, such as the kidney and inner ear stria vascularis^34^.

Previous studies have suggested that HL is associated with diabetes. One systematic study analyzed the relationship between diabetes and HL and aimed to conduct a quantitative and systematic review of the evidence regarding the effects of type 2 DM on hearing function. Patients with type 2 diabetes had a substantially higher incidence of mild HL than controls. The mean PTA thresholds were higher in patients with diabetes at all frequencies; however, results at 6000 Hz and 8000 Hz were more clinically relevant. Age and DM duration may play significant roles in the development of DM-related HL^35^. Teng et al.^36^ analyzed the relationship between the presence of type 1 DM and auditory dysfunction in their systematic review. They demonstrated that type 1 diabetes was associated with a higher risk of mild and subclinical hearing impairment. Although the types of DM were not categorized in this study, the mild HL observed in the DM group is similar to results of previous studies^35,36^.

Kim et al.^21^ evaluated the relationship between DM and the incidence of HL using data from a Hospital Health Screening Center. The multivariable-adjusted hazard ratios for HL in people with prediabetes or DM were 1.04 (95% CI 0.95–1.14) and 1.36 (1.19–1.56), respectively, compared to those with normal glucose levels. The risk of incident HL (pure-tone average of thresholds at 500, 1000, and 2000 Hz > 25 dB in both right and left ears) increased progressively with HbA1c levels greater than 5% in spline regression analyses. This previous study differs from the present study. First, the authors evaluated HL in the clinical audiogram range (500, 1000, and 2000 Hz) and did not include the 4000 Hz region that could explain the high-frequency range. Therefore, the distribution of hearing thresholds by group may differ in each study. The spline regression analyses of the previous study led the authors to suggest 5% HbA1c as a risk factor for HL. Diabetes can be diagnosed when HbA1c is 6.5% or higher; however, an HbA1c of 5% is within the normal range, making it challenging to confirm a link between diabetes and HL. Meanwhile, we investigated the HbA1c cutoff value that may indicate HL. To predict hearing impairment more than 25 dB, the ROC curve was examined, and a cutoff value of 6.3% HbA1c was found. An HbA1c of 6.3% is close to the level that would be categorized as diabetes; therefore, physicians should closely monitor hearing in patients with prediabetes.

Prediabetes is characterized as higher-than-normal blood glucose levels but not high enough to be classified as type 2 diabetes. However, without lifestyle changes, adults and children with prediabetes are at significant risk for developing type 2 diabetes. Prediabetes has been associated with long-term injury to the heart, blood vessels, and kidneys, even if type 2 diabetes has not yet developed^37^. A previous study showed that individuals with diabetes had twice the rate of HL than those without diabetes, and those with prediabetes had a 30% greater rate^38^. Through case–control analysis, our study enabled the identification of the risk of HL in the prediabetes and diabetes groups. The cases were categorized into distinct groups: the case group and control group, based on a PTA and pure-tone threshold of 25 dB for each frequency. Subsequently, adjusted OR values were determined by adjusting for age and sex. Upon examining the adjusted OR findings, there was no significant risk of hearing loss in the normal group and the prediabetes group. Conversely, there was a significant risk of hearing impairment at different frequencies when comparing those with normal HbA1c and hearing levels and those with diabetes. This indicates a significant correlation between elevated HbA1c and an increased risk of hearing impairment. The risk of HL increases if prediabetes progresses to diabetes; therefore, patients should be informed and aware of the need for management and treatment.

HL is the most common chronic health problem among older adults, and its age-specific incidence is increasing^2^. Hearing levels usually decline by approximately 1 dB per year after the age of 60 years. According to Lee et al.^39^, compared with younger females (60 to 69 years), older females (≥ 70 years) had a faster rate of change at 250 to 3000, 10,000, and 11,000 Hz. At 6000 Hz, hearing levels in older males changed more quickly than younger males. Compared with males, hearing levels in females changed more quickly between 6000 and 12,000 Hz and at 1000 Hz. HL typically occurs earlier in males than in females. SNHL is a progressive, symmetric HL, affecting more than 90% of older people with HL, primarily at high frequencies^40,41^. Many studies have reported an association between DM and high-frequency HL. A previous study suggested that mild HL is predominant in patients with diabetes and high-frequency hearing impairment^42^. Another study showed that patients with diabetes had higher mean PTA thresholds across all frequencies; however, those thresholds at 6000 and 8000 Hz were more clinically significant^35^.

This study had several limitations. As this was a cross-sectional study, we did not include information on the duration of the disease, treatment period and method, or other systemic causes of HL. Various blood parameters were not included in the study because of large variations among the subjects. Not considering or controlling for congenital hearing loss and ototoxic-drug exposure can be considered another limitation of our study. In addition, the population served by a tertiary care center does not inherently represent the general population. Our study population could differ substantially from the general population^43^. Furthermore, the average period between the two tests was approximately 35 days, with a standard deviation of approximately 28 days. However, this was a retrospective study and thus had the limitation that not all subjects were evaluated simultaneously.

In summary, this study showed that HL is associated with HbA1c levels. We found a correlation between HbA1c levels and hearing impairment. Screening for HL is strongly recommended for patients with elevated HbA1c levels. We expect this to be a very useful finding with important clinical implications when considering the care and treatment of DM in patients with HL. In the future, a large-scale prospective cohort study should be conducted to further examine changes in HbA1c and hearing levels in patients with DM.

## Data Availability

Any data generated and analyzed during this study that are not included in this published article and its additional information will be provided upon reasonable request to the corresponding author.
